# Antifungal therapy in patients with pulmonary *Candida* spp. colonization may have no beneficial effects

**DOI:** 10.1186/s40560-015-0097-0

**Published:** 2015-07-03

**Authors:** Simone Lindau, Manuel Nadermann, Hanns Ackermann, Tobias Michael Bingold, Christoph Stephan, Volkhard A. J. Kempf, Pia Herzberger, Andres Beiras-Fernandez, Kai Zacharowski, Patrick Meybohm

**Affiliations:** Department of Anesthesiology, Intensive Care Medicine and Pain Therapy, University Hospital Frankfurt, Frankfurt, Germany; Institute of Biostatistics and Mathematical Modelling, University Hospital Frankfurt, Frankfurt, Germany; Department of Medicine II: Infectiology, University Hospital Frankfurt, Frankfurt, Germany; Institute for Medical Microbiology and Infection Control, University Hospital Frankfurt, Frankfurt, Germany; Department of Thoracic and Cardiovascular Surgery, University Hospital Frankfurt, Frankfurt, Germany

**Keywords:** *Candida* spp. colonization, Antifungal therapy, Pneumonia

## Abstract

**Background:**

In critically ill patients, *Candida* spp. can often be identified in pulmonary samples. The impact of prompt antifungal therapy in these patients is unknown.

**Methods:**

In this retrospective study, 500 adult patients with pulmonary *Candida* spp. colonization admitted to the intensive care unit (ICU) between 2010 and 2012 were included. The patients were analyzed according to whether or not they received antifungal therapy, which was administered at the discretion of the attending physician. Logistic regression analysis was performed to investigate the impact of antifungal therapy on hospital mortality and new onset of ventilator-associated pneumonia. In a stepwise backward elimination, the impact of age, cancer as an underlying disease, Simplified Acute Physiology Score (SAPS) II, and Sequential Organ Failure Assessment (SOFA) score were considered.

**Results:**

After excluding 178 patients with multifocal *Candida* spp., isolated pulmonary *Candida* spp. colonization was found in 322 patients (cohort 1). Pre-existing pneumonia was found in 147/322 patients. Out of the remaining 175 patients (cohort 2), 44 patients received any antifungal therapy, and 131 were defined as the control group. Patients who received antifungal therapy had higher hospital mortality (50 vs. 30 %, *p* = 0.02) and pneumonia rates (47.7 vs. 16.8 %; *p* < 0.001) than those who did not. In Cox regression analysis, antifungal therapy was not independently associated with favorable outcome (mortality: odds ratio 0.854 (95 % CI 0.467–1.561); new pneumonia: 1.048 (0.536–2.046)), but SAPS II and SOFA score were significantly (*p* < 0.05) independent covariates for worse outcome.

**Conclusions:**

In critically ill patients with pulmonary *Candida* spp. colonization, antifungal therapy may not have an impact on the incidence of new pneumonia or in-hospital mortality after adjustment for confounders.

**Electronic supplementary material:**

The online version of this article (doi:10.1186/s40560-015-0097-0) contains supplementary material, which is available to authorized users.

## Background

Yeasts are part of the physiological flora of the oral mucosa and the intestine tract in about 40–65 % of healthy adults [1]. In immunosuppressed patients, however, yeasts can cause severe infections [2]. The same may be true for non-neutropenic critically ill patients, where yeasts can infrequently be found after a prolonged interval of medical illness with complex modulation of the immune system. This particularly refers to critically ill patients with ongoing intravascular catheters, prolonged antibiotic therapy, chemotherapy, or long-term ventilation [3]. In clinical routine, *Candida* spp. can very often be identified in pulmonary samples taken from tracheal aspirates or bronchoalveolar lavage. However, the key question remains whether a simple colonization or an invasive *Candida* spp. infection exists. Meersseman et al. recently published that 57 % of their deceased patients had findings of *Candida albicans* in the pulmonary secretion, but none of these patients had a “true” *Candida* spp. pneumonia [4]. For that reason, the European Society for Clinical Microbiology and Infectious Diseases (ESCMID) recommends that “*Candida* spp. isolation from respiratory secretions alone should never prompt treatment” [5].

In contrast, multifocal *Candida* spp. findings increase the risk of a systemic *Candida* spp. infection, and thereby increase risk for morbidity and mortality [6]. León et al. recently reported the *Candida* colonization index as a risk score for systemic *Candida* spp. infection including the status of (a) multifocal colonization, (b) surgery, (c) parenteral nutrition, and (d) severe sepsis [7].

Interestingly, Azoulay et al. found in a multicenter cohort of 800 patients that pulmonary *Candida* spp. colonization was significantly associated with an increased risk of nosocomial pneumonia and prolonged length of stay at the intensive care unit (ICU) [8]. Additionally, Hamet et al. reported in 300 critically ill patients with ventilator-associated pneumonia that pulmonary *Candida* spp. colonization was found in 56 % of these patients and represents an independent risk factor for multidrug-resistant bacterial super-infection associated with an increased risk of mortality [9].

In this respect, whether or not antifungal therapy should be initiated in critically ill patients with pulmonary *Candida* spp. colonization remains to be elucidated. In our retrospective study, we hypothesized that initiation of any antifungal therapy was associated with a reduced risk of new onset of ventilator-associated pneumonia and death compared to no antifungal therapy.

## Methods

This is a retrospective study including 500 intensive care patients with at least one pulmonary finding of *Candida* spp. The patients were treated between 2010 and 2012 at the intensive care unit of the Department of Anesthesiology, Intensive Care Medicine and Pain Therapy, University Hospital Frankfurt, Germany. This study was approved by the ethical committee of the Faculty of Medicine, University Hospital Frankfurt (379/12). The ethic committee did not have any ethical concerns and confirmed that no patient consent form is needed as this study retrospectively analyzed routine data.

As a clinical routine, pulmonary samples were taken as tracheal aspirates tracheal aspirates for microbiological analyses twice a week and additionally when a new pulmonary infection was considered (tracheal aspirates or bronchoalveolar lavage). The samples were created in laboratory natively and after dilution quantitatively on nutrient agar. The results were considered positive in the presence of *Candida* spp. growth in the culture medium. The different *Candida* isolates were identified at species level. Serologic biomarkers for *Candida* spp. were not used routinely. Indication for antifungal treatment was based on an individual decision considering the underlying risk factors of the critically ill patient, respectively. These factors particularly covered neutropenia, renal replacement therapy, long-term ventilation, or multifocal colonization.

### Endpoints

Primary endpoints were in-hospital mortality or any new onset of ventilator-associated pneumonia. Ventilator-associated pneumonia was defined as present (a) if the attending physician declared a new onset of a pulmonary infection and indicated broad-spectrum antibiotics or (b) if a new onset of pneumonia was defined according to the clinical and microbiological criteria of the CDC (Centers for Disease Control and Prevention) within the German Hospital Infection Surveillance System [10].

Secondary endpoints were length of stay at the intensive care unit, duration of ventilation based on the documented time of ventilation within the DRG coding, length of hospital stay, SAPS II (Simplified Acute Physiology Score), SOFA (Sequential Organ Failure Assessment) score at baseline and day 7 (without nervous system), and systemic inflammatory mediators at baseline and days 3, 7, and 14 including C-reactive protein, procalcitonin, leukocytes, thrombocytes, interleukin-6, and lipoprotein-binding protein. Also, we documented gender, primary discipline, cancer, any antibiotics and antifungal therapy, any pulmonary microbiology findings, and occurrence of sepsis and septic shock. Data collection was started as soon as the first positive microbiological finding with *Candida* spp. was reported (baseline). The observation time period for microbiological results and therapy was limited to a maximum of 28 days of ICU therapy.

### Sample size

This study has a retrospective observational design and is exploratory in nature and undertaken for description. Therefore, no direct comparisons between different antifungal strategies and *Candida* spp. have been performed.

For sample size calculation, we took into account previous study results. Risk of ventilator-associated pneumonia was significantly increased in patients with *Candida* spp. colonization (24 % out of 214 patients vs. 19 % [8] and 32 % out of 181 patients vs. 23 % [9]). Antifungal treatment reduced the risk of ventilator-associated pneumonia in 36 patients with *Candida* spp. colonization by 50 % [11]. We estimated that about 25 % of our total cohort (*n* = 500) with pulmonary *Candida* spp. colonization would have received any antifungal drug resulting in about 80 patients for the therapy group compared to 350 untreated patients (control group).

Based on the above-mentioned clinical studies, incidence of ventilator-associated pneumonia was assumed to be about 30 % in untreated patients with positive *Candida* spp. colonization (control group). We assumed that antifungal therapy reduces the incidence of pneumonia by 50 % with a level of significance of 0.05 and a power of 80 % by a two-sided test.

### Statistics

Metrically scaled variables were presented as mean ± standard deviation (SD) or median (25 %; 75 % quartile). The *p* values were determined by using the two-sided Wilcoxon-Mann-Whitney test. Nominally scaled variables were presented in absolute terms and percentage, based on the type of group. The *p* values were determined by using the Fisher-Yates test.

To investigate the impact of any antifungal therapy in pulmonary colonized patients on the incidence of pneumonia and on in-hospital mortality, we performed Kaplan-Meier analysis with Mantel-Cox logrank test for survival time and pneumonia-free time as the dependent variables. Additionally, we performed Cox regression analysis considering the impact of age, cancer as an underlying disease, SAPS II, and SOFA score (baseline values). The regression results are displayed as odds ratio (95 % confidence interval). The number of cases and these five predictor variables in Cox models stick to the rule of thumb, using around ten outcome events per predictor variable [12, 13]. In a final step, we performed a competing risk analysis (Aalen-Johansen estimator and logrank test) for pneumonia and death as competing events.

All statistics were two-tailed. Statistically significance was considered with a *p* < 0.05.

The data have been analyzed using IBM SPSS statistics 22 for Mac OS X and Bias 10.12.

## Results

**Fig. 1:**
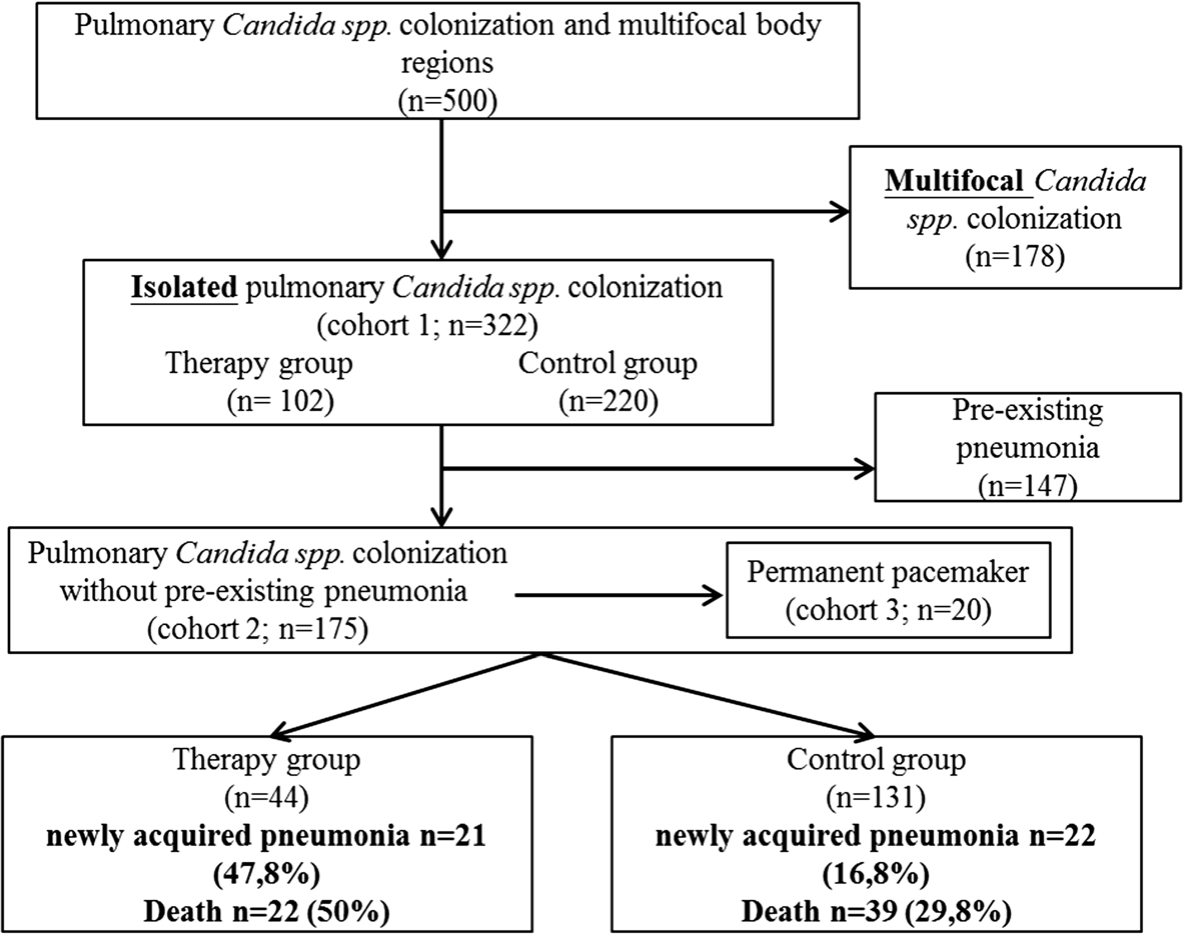
Flow chart illustrating the recruitment process. 500 patients were eligible for this study. Cohort 1: 322 patients with isolated pulmonary *Candida* spp. colonization with or without pre-existing pneumonia after excluding 178 patients with multifocal colonization. Cohort 2: 175 patients with isolated pulmonary *Candida* spp. colonization without any pre-existing pneumonia (therapy group: *n* = 44; control group: *n* = 131) after excluding 147 patients who had a pre-existing pneumonia before enrollment

Between 2010 and 2012, approximately 6000 patients were admitted to our ICU, of whom we analyzed 500 patients with any pulmonary finding of *Candida* spp. In the first step, we excluded patients with multifocal *Candida* spp. findings (*n* = 178), as multifocal colonization increases risk of systemic *Candida* spp. infection and triggers antifungal therapy. Isolated pulmonary *Candida* spp. colonization was found in 322 patients (cohort 1), of whom 102 patients received any antifungal therapy. Pre-existing pneumonia at baseline was found in 147/322 patients. Out of the remaining 175 patients (cohort 2), 44 patients did receive (therapy group) and 131 did not receive any antifungal therapy (control group; Fig. 1). To elucidate the role of antifungal therapy in high-risk patients of suspected candidemia, post hoc subgroup analysis was performed in patients with a permanent pacemaker placement (cohort 3).

### Cohort 1 (*n* = 322)

Baseline characteristics of patients with isolated pulmonary *Candida* spp. colonization including patients with pre-existing pneumonia are presented in Table 1 and Additional files 1 and 2. Most of the patients had cardiac surgery (*n* = 142). The patients with antifungal therapy were more critically ill with significantly (*p* < 0.001) higher SAPS II and SOFA scores, higher percentage of pre-existing pneumonia (56.9 vs. 40.5 %), higher use of carbapenems, quinolones, and glycopeptides, and higher incidence of *Candida non-albicans species* at baseline. Additional file 1 presents antimicrobial therapy. Antibiotics were given in 99 % of the patients in the therapy group (vs. 84.1 %) 39 out of 102 patients (38.2%) patients (38.2 %) already received antifungal therapy at baseline, and in the rest of the therapy group, antifungal therapy was started in median after 4 days (1; 6). The mean duration of antifungal therapy was 9 (5; 15) days. Additional file 2 presents microbiological findings before enrollment. Most common *Candida* spp. were *C. albicans* (69.6 vs. 81.8 %). Interestingly, significantly more therapy patients had any pulmonary bacterial findings at baseline (25 vs. 43 %, *p* = 0.002).

**Table 1:** Baseline characteristics of patients with isolated pulmonary *Candida* spp. colonization (cohort 1)

	Antifungal therapy (*n* = 102)	No antifungal therapy (*n* = 220)	*p* value
Age (years)	69 (57; 76)	70 (58/78)	0.354
Sex male, *n* (%)	83 (81.4)	162 (73.6)	0.16
Discipline, *n* (%)			
Thoracic and cardiovascular surgery	52 (50.1)	90 (40.9)	0.093
General and visceral surgery	12 (11.8)	33 (15.0)	0.493
Trauma surgery	9 (8.8)	29 (13.2)	0.353
Vascular surgery	12 (11.8)	25 (11.4)	1
Urology	3 (2.9)	1 (0.5)	0.096
Otolaryngology	0 (0)	5 (2.3)	0.183
Pneumology	3 (2.9)	4 (1.8)	0.683
Oral and maxillofacial surgery	0 (0)	4 (1.8)	0.311
Neurosurgery	3 (2.9)	20 (9.1)	0.061
Others	8 (7.8)	9 (4.1)	0.184
Cancer, *n* (%)	14 (13.7)	42 (19.1)	0.271
Permanent pacemaker, *n* (%)	15 (14.7)	17 (7.7)	0.071
SAPS II	47 (39; 55)	40 (30; 48)	<0.001
SOFA score	7 (5; 10)	4.5 (2; 8)	<0.001
Lung	2 (1; 2)	1 (1; 2)	0.437
Thrombocytes	1 (0; 2)	0 (0; 2)	0.015
Liver	0 (0; 2)	0 (0; 0)	<0.001
Cardiovascular	3 (0; 4)	0 (0; 3)	0.001
Renal	1 (0; 2)	0 (0; 2)	0.017
Pre-existing pneumonia before enrollment, *n* (%)	58 (56.9)	89 (40.5)	0.008

Data are presented as median (25 %; 75 % quartile)

*SAPS II* Simplified Acute Physiology Score II, *SOFA* Sequential Organ Failure Assessment

Outcome parameters are displayed in Table 2. In the therapy group, SOFA score on day 7 was higher (*p* < 0.005), 47.7 % of patients developed new pneumonia (vs. 16.8 %), durations of mechanical ventilation, hospital and ICU stay were longer (*p* < 0.001), more patients developed sepsis (*p* < 0.002) or septic shock (*p* < 0.006), and higher levels of pro-inflammatory mediators were found (*p* < 0.05; Additional file 3). Microbiological findings and therapy are summarized in Additional files 4 and 5.

**Table 2:** Outcome characteristics of patients with isolated pulmonary *Candida* spp. colonization (cohort 1)

	Antifungal therapy (*n* = 102)	No antifungal therapy (*n* = 220)	*p* value
SOFA score (day 7)	6 (3; 9)	3 (2; 7)	0.003
Lung	1 (1; 2)	1 (1; 2)	0.034
Thrombocytes	1 (0; 2)	0 (0; 2)	0.236
Liver	0 (0; 1)	0 (0; 0)	0.001
Cardiovascular	0 (0; 3)	0 (0; 3)	0.033
Renal	1 (0; 2)	1 (0; 2)	0.159
New pneumonia, *n* (%)	21 (20.6)	22 (10)	0.013
Sepsis, *n* (%)			
Sepsis/severe sepsis	27 (26.5)	26 (11.8)	0.002
Septic shock	29 (28.4)	32 (14.5)	0.006
Duration of mechanical ventilation (h)	362 (199; 599)	160 (67; 284)	<0.001
Hospital stay (days)	31 (17; 53)	21 (12; 35)	<0.001
ICU stay (days)	20 (11; 29)	8 (4; 14)	<0.001
In-hospital mortality, *n* (%)	53 (52.0)	76 (34.5)	0.003
Discharge from hospital, *n* (%)			
Other hospital	24 (23.5)	47 (21.4)	0.667
Rehabilitation clinic	19 (18.6)	42 (19.1)	1
Home	6 (5.9)	49 (22.3)	<0.001
Others	0 (0)	6 (2.7)	0.032

Data are presented as median (25 %; 75 % quartile)

SOFA Sequential Organ Failure Assessment, *ICU* intensive care unit

**Fig. 2:**
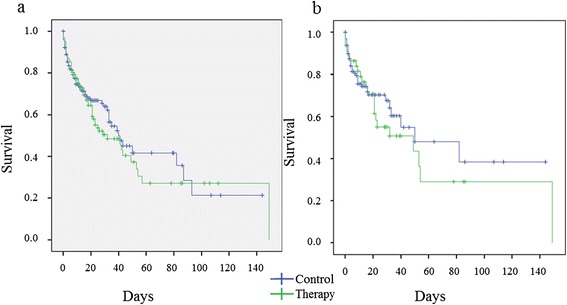
Kaplan-Meier analysis for survival time. **a** Cohort 1: patients with isolated pulmonary *Candida* spp. colonization (*n* = 322) including patients with pre-existing pneumonia; Mantel-Cox logrank test: *p* > 0.05. **b** Cohort 2: patients with isolated pulmonary *Candida* spp. colonization (*n* = 175) excluding patients with pre-existing pneumonia before enrollment; Mantel-Cox Logrank test: *p* > 0.05. Therapy group (*green line*) vs. control group (*blue line*)

Using the Kaplan-Meier estimate, survival curves did not show any significant difference between the groups (*p* > 0.05; Fig. 2a). Cox regression analysis revealed SAPS II (1.028 (1.008–1.048); *p* = 0.005), SOFA score (1.094 (1.030–1.163); *p* = 0.004), and age (1.020 (1.004–1.037); *p* = 0.016) as independent covariates for mortality, but antifungal therapy had no independent impact on chance of survival (1.198 (0.810–1.771); *p* = 0.366; Additional file 6).

### Cohort 2 (*n* = 175)

Focusing on patients with isolated pulmonary *Candida* spp. colonization without pre-existing pneumonia, baseline characteristics are presented in Table 3. SAPS II (*p* < 0.001) and SOFA score were significantly higher in the therapy group (*p* < 0.003). Additional file 7 presents microbiological findings and therapy. In 17 of 44 patients (38.6 %), preemptive antifungal therapy was already administered at baseline. Outcome parameters of the cohort 2 are presented in Table 4. In the therapy group, more patients became septic (40.9 vs. 20.6 %), had longer hospital (*p* < 0.005) and ICU (*p* < 0.001) stays, a higher incidence of newly acquired pneumonia (*p* < 0.001), and died more often (50 vs. 29.8 %; *p* = 0.02). During the observation period, 52.3 % had any evidence of pulmonary bacterial colonization/infection. Gram-negative bacteria were the most common co-infection (Additional file 8).

**Table 3:** Baseline characteristics of patients with isolated pulmonary *Candida* spp. colonization without pre-existing pneumonia (cohort 2)

	Antifungal therapy (*n* = 44)	No antifungal therapy (*n* = 131)	*p* value
Age (years)	70 (59; 76)	71 (56; 79)	0.57
Sex male, *n* (%)	38 (86.4)	94 (71.8)	0.07
Cancer, *n* (%)	5 (11.4)	24 (18.3)	0.35
Permanent pacemaker, *n* (%)	8 (18.2)	12 (9.1)	0.17
SAPS II	48 (37; 55)	37 (26; 47)	<0.001
SOFA score	7 (4; 10)	4 (2; 8)	0.003

Data are presented as median (25 %; 75 % quartile)

*SAPS II* Simplified Acute Physiology Score II, *SOFA* Sequential Organ Failure Assessment

**Table 4:** Outcome characteristics of patients with isolated pulmonary *Candida* spp. colonization without pre-existing pneumonia (cohort 2)

	Antifungal therapy (*n* = 44)	No antifungal therapy (*n* = 131)	*p* value
SOFA score (day 7)	7 (4; 10)	3 (1; 6)	0.003
Newly acquired pneumonia, *n* (%)	21 (47.7)	22 (16.8)	<0.001
Sepsis/severe sepsis, *n* (%)	18 (40.9)	27 (20.6)	0.01
Mechanical ventilation (h)	162 (140; 547)	152 (59; 242)	<0.001
Hospital stay (days)	31 (15; 53)	20 (10; 34)	0.004
ICU stay (days)	17 (10; 29)	7 (4; 12)	<0.001
In-hospital mortality, *n* (%)	22 (50)	39 (29.8)	0.02

Data are presented as median (25 %; 75 %) quartile

*SOFA* Sequential Organ Failure Assessment

**Fig. 3:**
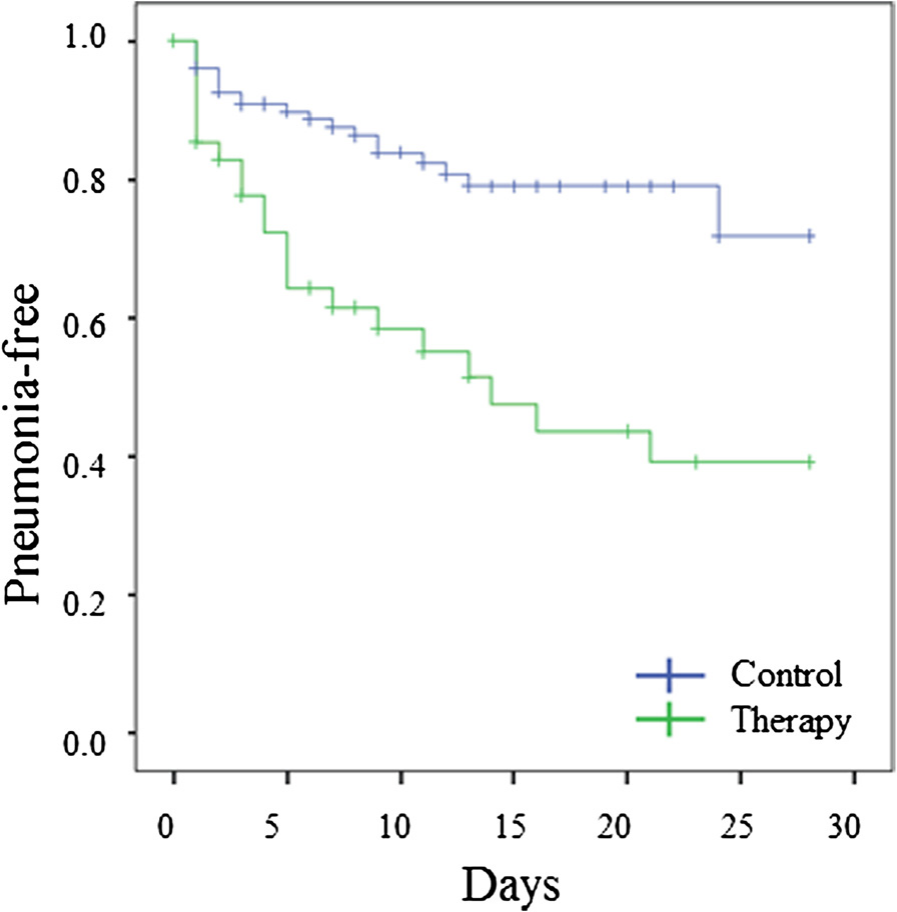
Kaplan-Meier analysis for time until new ventilator-associated pneumonia. Cohort 2: Patients with isolated pulmonary *Candida* spp. colonization without any pre-existing pneumonia before enrollment (*n* = 175); Mantel-Cox logrank test: *p* < 0.001. Therapy group (*green line*) vs. control group (*blue line*)

Kaplan-Meier estimate did not show a significant difference in the survival curves between the therapy and control group (*p* > 0.05; Fig. 2b). Using the Cox regression analysis, antifungal therapy had no significant independent impact on survival (0.855 (0.469–1.558), *p* = 0.608), but SAPS II (1.027 (1.003–1.052), *p* = 0.025) and SOFA score (1.096 (1.004–1.198), *p* = 0.041) were independently associated with higher risk of death (Additional file 9). The time until new hospital-acquired pneumonia was significantly shorter in the therapy group (*p* < 0.001; Fig. 3), but again, antifungal therapy had no significant impact using Cox regression analysis (1.034 (0.546–1.957), *p* = 0.919; Additional file 10).

In the final step, competing risk analysis with the Aalen-Johansen estimator for pneumonia and death as the competing events was performed. Again, the time until new event was significantly shorter in the therapy group (*p* < 0.001) (not shown).

### Cohort 3 (*n* = 20)

In case that blood culture shows false negative candidemia, we defined a subgroup of high-risk patients of suspected candidemia who had a permanent pacemaker placement (cohort 3). The baseline characteristics are presented in Table 5. SAPS II and SOFA score did not significantly differ between the groups. Mortality rate did not differ significantly (therapy group 4/8 (50 %) vs. control group 6/12 (50 %)). Using the Cox regression analysis, antifungal therapy had no significant independent impact on survival (1.693 (0.396–7.230), *p* = 0.477). The rate of new hospital-acquired pneumonia did not differ significantly (therapy group 2/8 (25 %) vs. control group 3/12 (25 %)). Antifungal therapy had no significant impact using Cox regression analysis (0.448 (0.11–18.71), *p* = 0.673).

**Table 5:** Baseline characteristics of high-risk patients with permanent pacemaker and isolated pulmonary *Candida* spp. colonization without pre-existing pneumonia (cohort 3)

	Antifungal therapy (*n* = 8)	No antifungal therapy (*n* = 12)	*p* value
Age (years)	73 (65; 79)	74 (69; 83)	0.69
Cancer, *n* (%)	0 (0)	4 (30)	0.12
SAPS II	50 (46; 56)	34 (29; 58)	0.09
SOFA score	10 (5; 11)	4 (3; 7)	0.08

Data are presented as median (25 %; 75 % quartile)

*SAPS II* Simplified Acute Physiology Score II, *SOFA* Sequential Organ Failure Assessment

## Discussion

*Candida* is frequently found in pulmonary secretions of patients in ICU known as *Candida* spp. colonization, if clinical and pathological evidence of invasive candidiasis is absent. In this respect, *Candida* spp. colonization may not play an important pathogenic role in the context of ventilator-associated pneumonia, at least in immunocompetent patients [4]. Consequently, the European Society for Clinical Microbiology and Infectious Diseases (ESCMID) do not recommend a systemic antifungal therapy based on isolated findings of *Candida* spp. in pulmonary secretion [5].

On the other hand, pulmonary *Candida* spp. colonization has been shown to be significantly associated with an increased risk of nosocomial pneumonia [8]. Additionally, pulmonary *Candida* spp. colonization is suggested to be an independent risk factor for multidrug-resistant bacterial super-infection associated with an increased risk of mortality [9]. In this respect, any therapeutic intervention to attenuate fungal pathogens might be an interesting option, at least in theory. In clinical routine, the individual decision to treat or not to treat is very difficult, and patients often receive an antifungal drug at the discretion of the individual attending physician irrespective of the guidelines.

Very recently, Albert et al. included 60 patients into a randomized trial and analyzed 29 patients within an observational study, whether antifungal therapy in critically ill patients with a clinical suspicion of ventilator-associated pneumonia with positive airway secretion specimens for *Candida* spp. might be beneficial [12]. Markers of inflammation and all clinical outcomes were comparable between placebo and antifungal treatment group at baseline and over time. These authors concluded that this study did not provide evidence to support a larger trial examining the efficacy of empiric antifungal treatment in patients with a clinical suspicion of ventilator-associated pneumonia and *Candida* spp. in the tracheal secretions.

The main results of our retrospective study including 322 critically ill patients are in line with these recent findings. Using Cox regression analysis to account for confounders, any antifungal treatment did not had a beneficial effect on the incidence of new pneumonia or in-hospital mortality in a cohort of heterogenic intensive care patients with isolated pulmonary *Candida* spp. colonization. Not surprisingly, patients that received antifungal therapy were more critically ill as evident from higher SAPS II and SOFA score and had a higher degree of systemic inflammation. In this respect, these patients showed prolonged duration of mechanical ventilation, ICU, and hospital length of stay.

Consistently, our Cox regression analysis revealed SAPS II, SOFA score, and age of patient as independent variables for a higher risk of in-hospital mortality. These results are not surprising, as SAPS II “…provides an estimate of the risk of death without having to specify a primary diagnosis” [14], and the SOFA score reasonably describes organ dysfunction in critically ill patients [15]. Nfor et al. evaluated a direct relationship between the total SOFA score at admission and ICU mortality [16]. Moreno et al. showed that ICU mortality was more strongly associated with ‘delta’ SOFA score, which is defined as the total maximum SOFA minus the SOFA score at admission [17].

In a subgroup of patients (cohort 2), we focused on the time until new pneumonia. If any antifungal therapy is suggested to be beneficial, then free time to any new pneumonia should reasonably be longer, at least in theory. Surprisingly, our results showed contradictory effects, and time until new pneumonia was significantly shorter in patients that received antifungal therapy during ICU stay. This is in line with our findings that patients with antifungal therapy were more critically ill with significantly higher incidence of new pneumonia, higher SAPS II and SOFA score, higher incidence of sepsis, and longer duration of hospital and ICU stays. Thus, severity of critical illness may have a large bias on outcome variables. To account for these confounders, we considered a couple of known covariates for pneumonia in our Cox regression analysis. Nevertheless, antifungal therapy may not have an independent effect on time to new pneumonia or survival chance. There are three additional interesting observations: (a) most of the patients developed new pneumonia within the first 10 days after microbiological detection of yeasts, (b) preemptive antifungal therapy has been observed in 38 % of the patients, and (c) median time to initiate antifungal therapy was 4 days in non-preemptive-treated patients. Under these circumstances, it remains speculative if a faster initiation of antifungal therapy at the first day of microbiological result or even a more preemptive antifungal strategy in critically ill patients would otherwise prevent any yeasts and would result in a better outcome. Nevertheless, these factors are of minor importance regarding the key question of this analysis—whether or not to initiate antifungal therapy in critically ill patients with *Candida* spp. colonization.

Our results also suggest that the absence of any beneficial effect after antifungal therapy is irrespective of any pre-existing pneumonia. Thus, even in higher risk critically ill patients with pre-existing ventilator-associated pneumonia (cohort 1), the presence of *Candida* spp. in the lung could be interpreted as harmless colonization rather than representing a true infection requiring any treatment [18]. Very recently, Blot et al. found several risk factors for mortality in patients with ventilator-associated pneumonia, e.g., diabetes and septic shock [19], but similarly *Candida* spp. colonization had no significant impact in this study.

Thus, our results support the recent ESCMID recommendations, not to initiate antifungal therapy in patients with isolated pulmonary *Candida* spp. colonization irrespective of the severity of critical illness.

Importantly, blood culture may show false negative results of candidemia among high-risk patients with central venous catheter or permanent pacemaker placement due to relatively slow growth of *Candida* spp. in the blood culture. To elucidate the role of antifungal therapy in these high-risk patients, we performed a subgroup analysis of 20 patients with implanted pacemaker and isolated pulmonary *Candida* spp. colonization without pre-existing pneumonia. Again, antifungal therapy was not independently associated with a favorable outcome.

Our study has the following limitations. Firstly, this study has a retrospective and explorative design, and a large prospective randomized controlled study might be needed to provide more evidence whether or not a fungal therapy may be beneficial in patients with isolated pulmonary *Candida* spp. colonization. Secondly, we did not consider additional risk factors for ventilator-associated pneumonia as covariates, e.g., chronic obstructive pulmonary disease [20], the use of broad-spectrum antibiotics in the preceding days [21], diabetes mellitus [22], and the use of proton pump inhibitors [23], as these parameters would not be validly available using a retrospective design in our hospital. Finally, it is well known that different *Candida* spp. *(albicans*, *krusei*, etc.) have different pathogenicity and sensibilities to drugs, but we did not perform subgroup analyses regarding different types of *Candida* spp. or kinds of antifungal drugs, as the sample size is reasonable small.

## Conclusions

Our study suggests that antifungal therapy may not have an impact on the incidence of new pneumonia or in-hospital mortality after adjustment for confounders in critically ill patients with isolated pulmonary *Candida* spp.

## Additional files

Additional file 1:
**Baseline microbiological therapy in patients with isolated pulmonary** ***Candida*** **spp. colonization (cohort 1).** Different antifungal drugs and antibiotics that were given at baseline are shown. Additional file 2:
**Baseline pulmonary microbiological findings in patients with isolated pulmonary** ***Candida*** **spp. colonization (cohort 1).|** *Candida* spp., Aspergillus and any bacterial findings at baseline are shown. Additional file 3:
**Inflammatory mediators in patients with isolated pulmonary** ***Candida*** **spp. colonization (cohort 1).** Different inflammatory mediators are shown on day 1, 3, 7 and 14. Additional file 4:
**Microbiological therapy in patients with isolated pulmonary** ***Candida*** **spp. colonization during observation period (cohort 1).** Different antifungal drugs and antibiotics that were given during observation period are shown. Additional file 5:
**Pulmonary microbiological findings in patients with isolated pulmonary** ***Candida*** **spp. colonization during observation period (cohort 1).** *Candida* spp., Aspergillus and any bacterial findings during observation period are shown. Additional file 6:
**Patients with isolated pulmonary** ***Candida*** **spp. colonization—stepwise backwards elimination for survival time (cohort 1).** Cox regression analysis for independent impact on survival was performed for potential co-variable (Therapy, SAPS II, SOFA score, Age and Cancer). Additional file 7:
**Baseline pulmonary microbiological findings and therapy in patients with isolated pulmonary** ***Candida*** **spp. colonization without pre-existing pneumonia (cohort 2).** Different antifungal drugs and antibiotics that were given and *Candida* spp., Aspergillus and any bacterial findings at baseline are shown. Additional file 8:
**Pulmonary microbiological findings and therapy in patients with isolated pulmonary** ***Candida*** **spp. colonization without pre-existing pneumonia during observation period (cohort 2).** Different antifungal drugs and antibiotics that were given and *Candida* spp., Aspergillus and any bacterial findings during observation period are shown. Additional file 9:
**Patients with isolated pulmonary** ***Candida*** **spp. colonization without pre-existing pneumonia—stepwise backwards elimination for survival time (cohort 2).** Cox regression analysis for independent impact on survival was performed for potential co-variable (Therapy, SAPS II, SOFA score, Age and Cancer). Additional file 10:
**Patients with isolated pulmonary** ***Candida*** **spp. colonization without pre-existing pneumonia—stepwise backwards elimination for pneumonia-free time (cohort 2).** Cox regression analysis for independent impact on newly aquired pneumonia was performed for potential co-variable (Therapy, SAPS II, SOFA score, Age and Cancer).

## Abbreviations

ESCMID: European Society for Clinical Microbiology and Infectious Diseases
ICU: intensive care unit
MDR: multidrug resistance
SAPS: Simplified Acute Physiology Score
SOFA: Sequential Organ Failure Assessment
