# Supplementary material for: Antifungal therapy in patients with pulmonary Candida spp. colonization may have no beneficial effects
Source: J Intensive Care. 2015 Jul 3;3(1):31. doi: 10.1186/s40560-015-0097-0 (PMC4490727; doi:10.1186/s40560-015-0097-0)
Supplement: Additional file 1: — Baseline microbiological therapy in patients with isolated pulmonary Candida spp. colonization (cohort 1). Different antifungal drugs and antibiotics that were given at baseline are shown. [file 40560_2015_97_MOESM1_ESM.pdf]

Additional file 1. Baseline microbiological therapy in patients with isolated pulmonary *Candida spp.* colonization (cohort 1).

	**Antifungal therapy (n=102)**	**No antifungal therapy (n=220)**	**p-value**
**Antifungal drugs,** n (%)			
Echinocandins	16 (15.7%)		
Fluconazole	19 (18.6%)		
Others[1]	4 (3.9%)		
Combined[2]	1 (1%)		
None	63 (61.8%)		
**Antibiotics,** n (%)			
Aminoglycosides	2 (2%)	8 (3.6%)	0.113
Penicillin	21 (20.6%)	55 (25%)	0.402
Cephalosporins	25 (24.5%)	39 (17.7%)	0.177
Quinolones	31 (30.4%)	38 (17.3%)	**0.009**
Macrolides	1 (1%)	9 (4.1%)	0.179
Carbapenems	37 (36.3%)	64 (29.1%)	0.2
Glycopeptides	46 (45.1%)	58 (26.4%)	**0.001**
Linezolid	9 (8.8%)	6 (2.7%)	**0.022**
Daptomycin	1 (1%)	1 (0.5%)	0.534
Others[3]	16 (15.7%)	19 (8.6%)	0.082

[1] Amphotericin B, Voriconazole.
[2] at least two different antifungal drugs.
[3] Chloramphenicol, Cotrimoxazole, Doxycycline, Fosfomycin, Fusidic acid, Metronidazole, Quinupristin/Dalfopristin, Rifampicin, Tigecycline, Trimethoprim/Sulfamethoxazole.
