# Supplementary material for: Antifungal therapy in patients with pulmonary Candida spp. colonization may have no beneficial effects
Source: J Intensive Care. 2015 Jul 3;3(1):31. doi: 10.1186/s40560-015-0097-0 (PMC4490727; doi:10.1186/s40560-015-0097-0)
Supplement: Additional file 2: — Baseline pulmonary microbiological findings in patients with isolated pulmonary Candida spp. colonization (cohort 1).| Candida spp., Aspergillus and any bacterial findings at baseline are shown. [file 40560_2015_97_MOESM2_ESM.pdf]

Additional file 2. Baseline pulmonary microbiological findings in patients with isolated pulmonary *Candida spp.* colonization (cohort 1).

	**Antifungal therapy (n=102)**	**No antifungal therapy (n=220)**	**p-value**
Candida spp., n (%)			
albicans	71 (69.6%)	180 (81.8%)	**0.02**
glabrata	18 (17.6%)	37 (16.8%)	0.874
tropicalis	11 (10.8%)	15 (6.8%)	0.271
krusei	6 (5.9%)	1 (0.5%)	**0.005**
Others[1]	8 (7.8%)	9 (4.1%)	0.184
Co-Infection with *Aspergillus,* n (%)	2 (2%)	3 (1.4%)	0.654
**Any pulmonary bacterial finding,** n (%)	25 (25%)	94 (43%)	**0.002**
Gram positive	6 (5.9%)	17 (7.7%)	0.647
Gram negative	21 (20.6%)	84 (38.2%)	**0.002**
E. coli	5 (4.9%)	21 (9.5%)	0.19
Pseudomonas aeruginosa	5 (4.9%)	14 (6.4%)	0.8
Klebsiella pneumonia	3 (2.9%)	9 (4.1%)	0.759
Enterobacter species	3 (2.9%)	9 (4.1%)	0.759
Others[2]	5 (4.9%)	31 (14.1%)	**0.014**
multi drug resistant pathogens	2 (2%)	5 (2.3%)	1

[1] *Candida famata, kefyr, lipolytica, lusitaniae* and *parapsilosis.*

[2] *Stenotrophomonas, Proteus mirabilis, Serratia marcescens, Citrobacter koseri, Actinetobacter baumanii, Klebsiella ocytoca, Proteus vulgaris, Citrobacter freundii, Morganella morganii.*
