# Supplementary material for: Antifungal therapy in patients with pulmonary Candida spp. colonization may have no beneficial effects
Source: J Intensive Care. 2015 Jul 3;3(1):31. doi: 10.1186/s40560-015-0097-0 (PMC4490727; doi:10.1186/s40560-015-0097-0)
Supplement: Additional file 3: — Inflammatory mediators in patients with isolated pulmonary Candida spp. colonization (cohort 1). Different inflammatory mediators are shown on day 1, 3, 7 and 14. [file 40560_2015_97_MOESM3_ESM.pdf]

Additional file 5. Inflammatory mediators in patients with isolated pulmonary *Candida spp.* colonization (cohort 1).

	**Antifungal therapy (n=102)**	**No antifungal therapy (n=220)**	**p-value**
**Leucocytes**			
day 1 (n=321)	14.3 (10.2;19.7)	12.4 (8.7;17.1)	**0.02**
day 3 (n=249)	16.4 (12;21.5)	12.8 (9.4;17.8)	**<0.001**
day 7 (n=308)	16 (11.1;21.7)	12.8 (9.6;17.4)	**0.004**
day 14 (n=84)	13.7 (10;16)	11.7 (7.9;14.5)	0.14
**PCT**			
day 1 (n=305)	2 (1;7)	1 (0;4)	**0.024**
day 3 (n=243)	2 (1;9)	1 (0;3)	**<0.001**
day 7 (n=164)	1 (1;4)	1 (0;2)	**0.004**
day 14 (n=83)	1 (1;2)	1 (0;1)	0.131
**CRP**			
day 1 (n=314)	15.3 (9.6;25.2)	13.1 (7;22)	0.09
day 3 (n=241)	14.8 (8.9;23)	11.7 (8;21)	0.087
day 7 (n=153)	9.9 (6.1;16)	9 (6;14)	0.389
day 14 (n=82)	9.5 (5.5;15.4)	9.1 (5;14)	0.625
**Interleukin-6**			
day 1 (n=225)	174 (72;548)	101 (52;303)	**0.041**
day 3 (n=192)	96 (47;263)	62 (30;148)	**0.006**
day 7 (n=106)	78 (35;212)	49 (22;103)	**0.023**
day 14 (n=52)	91 (48;212)	58 (29;124)	0.118
**LBP**			
day 1 (n=216)	23 (16;35)	24 (15;32)	0.917
day 3 (n=185)	23 (16;36)	22 (15;34)	0.826
day 7 (n=102)	17 (10;28)	17 (10;28)	0.76
day 14 (n=50)	17 (11;26)	18 (12;24)	1
**Thrombocytes**			
day 1 (n=321)	116 (66;200)	154 (92;223)	**0.013**
day 3 (n=249)	113 (63;227)	150 (92;245)	**0.049**
day 7 (n=164)	146 (87;255)	204 (87;316)	0.127
day 14 (n=84)	161 (89;288)	213 (123;187)	0.061

Data are presented in median and (25%/75%) quartile. PCT – procalcitonin, CRP – c-reactive-protein, LBP – lipopolysaccharide-binding protein.
