# Supplementary material for: Antifungal therapy in patients with pulmonary Candida spp. colonization may have no beneficial effects
Source: J Intensive Care. 2015 Jul 3;3(1):31. doi: 10.1186/s40560-015-0097-0 (PMC4490727; doi:10.1186/s40560-015-0097-0)
Supplement: Additional file 4: — Microbiological therapy in patients with isolated pulmonary Candida spp. colonization during observation period (cohort 1). Different antifungal drugs and antibiotics that were given during observation period are shown. [file 40560_2015_97_MOESM4_ESM.pdf]

Additional file 3. Microbiological therapy in patients with isolated pulmonary *Candida spp.* colonization during observation period (cohort 1).

	Antifungal therapy (n=102)	No antifungal therapy (n= 220)	p-value
**Antifungal drugs**, n (%)			
Echinocandins	51 (50%)		
Fluconazole	56 (54.9%)		
Others[1]	17 (16.7%)		
Combined[2]	21 (20.6%)		
**Start of antifungal therapy (days)**	4 (1;6)		
**Duration antifungal therapy (days)**	9 (5;15)		
**Antibiotics,** n (%)	101 (99%)	185 (84.1%)	**<0.001**
Aminoglycosides	8 (7.8%)	8 (3.6%)	0.165
Penicillin	40 (39.2%)	65 (29.5%)	0.097
Cephalosporins	18 (17.6%)	47 (21.4%)	0.461
Quinolones	66 (64.7%)	61 (27.7%)	**<0.001**
Macrolides	2 (2%)	10 (4.5%)	0.352
Carbapenems	67 (65.7%)	81 (36.8%)	**<0.001**
Glycopeptides	67 (65.7%)	67 (30.5%)	**<0.001**
Linezolid	23 (22.5%)	9 (4.1%)	**<0.001**
Daptomycine	5 (4.9%)	1 (0.5%)	**0.013**
Others[3]	30 (29.4%)	22 (10%)	**<0.001**

Data are presented as median (25%;75% quartile).

[1] Amphotericin B, Voriconazole.
[2] at least two different antifungal drugs.
[3] Chloramphenicol, Cotrimoxazole, Doxycycline, Fosfomycin, Fusidic acid, Metronidazole, Quinupristin/Dalfopristin, Rifampicin, Tigecycline, Trimethoprim/Sulfamethoxazole.
