# Supplementary material for: Antifungal therapy in patients with pulmonary Candida spp. colonization may have no beneficial effects
Source: J Intensive Care. 2015 Jul 3;3(1):31. doi: 10.1186/s40560-015-0097-0 (PMC4490727; doi:10.1186/s40560-015-0097-0)
Supplement: Additional file 5: — Pulmonary microbiological findings in patients with isolated pulmonary Candida spp. colonization during observation period (cohort 1). Candida spp., Aspergillus and any bacterial findings during observation period are shown. [file 40560_2015_97_MOESM5_ESM.pdf]

Additional file 4. Pulmonary microbiological findings in patients with isolated pulmonary *Candida spp.* colonization during observation period (cohort 1).

	**Antifungal therapy (n=102)**	**No antifungal therapy (n=220)**	**p-value**
Candida spp., n (%)			
albicans	78 (76.5%)	183 (83.2%)	0.17
glabrata	31 (30.4%)	42 (19.1%)	**0.031**
tropicalis	16 (15.7%)	18 (8.2%)	0.051
krusei	6 (5.9%)	1 (0.5%)	**0.005**
Others[1]	15 (14.7%)	23 (10.5%)	0.271
Co-Infection with *Aspergillus*, n (%)	2 (2%)	3 (1.4%)	0.654
**Any new pulmonary bacterial finding,** n (%)	53 (52%)	120 (55%)	0.719
Gram positive	15 (14.7%)	25 (11.4%)	0.468
Gram negative	48 (47.1%)	107 (48.6%)	0.811
E. Coli	11 (10.8%)	25 (11.4%)	1
Pseudomonas aeruginosa	12 (11.8%)	15 (6.8%)	0.193
Klebsiella pneumonia	8 (7.8%)	14 (13.7%)	0.639
Enterobacter species	8 (7.8%)	13 (5.9%)	0.628
Others[2]	9 (8.8%)	40 (18.2%)	**0.031**
multi drug resistant pathogens	6 (5.9%)	7 (3.2%)	0.36

[1] *Candida famata, kefyr, lipolytica, lusitaniae* and *parapsilosis.*
[2] *Stenotrophomonas, Proteus mirabilis, Serratia marcescens, Citrobacter koseri, Actinetobacter baumanii, Klebsiella ocytoca, Proteus vulgaris, Citrobacter freundii, Morganella morgannii, Burkholderia cepacia.*
