# Supplementary material for: Antifungal therapy in patients with pulmonary Candida spp. colonization may have no beneficial effects
Source: J Intensive Care. 2015 Jul 3;3(1):31. doi: 10.1186/s40560-015-0097-0 (PMC4490727; doi:10.1186/s40560-015-0097-0)
Supplement: Additional file 6: — Patients with isolated pulmonary Candida spp. colonization—stepwise backwards elimination for survival time (cohort 1). Cox regression analysis for independent impact on survival was performed for potential co-variable (Therapy, SAPS II, SOFA score, Age and Cancer). [file 40560_2015_97_MOESM6_ESM.pdf]

Additional file 6. Patients with isolated pulmonary *Candida spp.* colonization - stepwise backwards elimination for survival time (cohort 1).

		**Exp(B)**	**95% CI for Exp(B)**		**B**	**Wald**	**Sign.**
			**Lower bound**	**Upper bound**			
**Step 1**	Therapy	1.198	0.810	1.771	0.180	0.0817	0.366
	SAPS II	1.030	1.010	1.050	0.029	8.764	0.003
	SOFA score	1.098	1.033	1.168	0.094	8.905	0.003
	Age	1.019	1.002	1.035	0.018	4.847	0.028
	Cancer	1.298	0.835	2.017	0.261	1.341	0.247
**Step 2**	SAPS II	1.028	1.009	1.048	0.028	8.085	0.004
	SOFA score	1.095	1.030	1.164	0.091	8.468	0.004
	Age	1.019	1.003	1.036	0.019	5.281	0.022
	Cancer	1.336	0.863	2.066	0.289	1.691	0.193
**Step 3**	SAPS II	1.028	1.008	1.048	0.028	7.983	**0.005**
	SOFA score	1.094	1.030	1.163	0.090	8.450	**0.004**
	Age	1.020	1.004	1.037	0.020	5.855	**0.016**

Exp(B) – odds ratio, CI – confidence interval, B – Not standardized regression coefficient, Wald – Wald-statistics, Sign. –Significance (p-value). SAPS II - Simplified Acute Physiology Score II ($p<0.005$), SOFA - Sequential Organ Failure Assessment ($p<0.005$) and Age ($p<0.05$) are independent variables that significantly influence the dependent variable (survival time).
