# Supplementary material for: Antifungal therapy in patients with pulmonary Candida spp. colonization may have no beneficial effects
Source: J Intensive Care. 2015 Jul 3;3(1):31. doi: 10.1186/s40560-015-0097-0 (PMC4490727; doi:10.1186/s40560-015-0097-0)
Supplement: Additional file 7: — Baseline pulmonary microbiological findings and therapy in patients with isolated pulmonary Candida spp. colonization without pre-existing pneumonia (cohort 2). Different antifungal drugs and antibiotics that were given and Candida spp., Aspergillus and any bacterial findings at baseline are shown. [file 40560_2015_97_MOESM7_ESM.pdf]

Additional file 7. Baseline pulmonary microbiological findings and therapy in patients with isolated pulmonary *Candida spp.* colonization without pre-existing pneumonia (cohort 2).

	**Antifungal therapy (n=44)**	**No antifungal therapy (n=131)**	**p-value**
Candida spp., n (%)			
albicans	30 (68.2%)	112 (85.5%)	**0.015**
glabrata	7 (15.9%)	20 (15.3%)	1
tropicalis	4 (9.1%)	6 (4.6%)	0.273
krusei	3 (6.8%)	1 (1%)	**0.05**
others[1]	3 (6.8%)	3 (2.3%)	0.168
Co-Infection with *Aspergillus,* n (%)	1 (2.3%)	1 (0.8%)	0.441
**Any pulmonary bacterial finding,** n (%)	8 (18.2%)	53 (40.5%)	**0.01**
Gram positive	3 (6.8%)	13 (9.9%)	0.764
Gram negative	7 (15.9%)	45 (34.4%)	**0.022**
E. Coli	3 (6,8%)	9 (6,9%)	1
Pseudomonas aeruginosa	0 (0%)	7 (5,3%)	0.194
Klebsiella pneumonia	1 (2,3%)	5 (3,8%)	1
Enterobacter species	0 (0%)	4 (3,1%)	0.573
Others[2]	3 (6,8%)	20 (15,3%)	0.2
multi drug resistant pathogens	0 (0%)	1 (0.8%)	1
**Antifungal treatment**, n (%)	17 (38.6%)		
**Antibiotic treatment**, n (%)	42 (96%)	92 (70.2%)	**<0.001**

[1] *Candida famata, lusitaniae* and *parapsilosis.*
[2] *Stenotrophomonas, Proteus mirabilis, Serratia marcescens, Citrobacter koseri, Actinetobacter baumanii, Proteus vulgaris, Citrobacter freundii, Morganella morgannii,*
