# Supplementary material for: Antifungal therapy in patients with pulmonary Candida spp. colonization may have no beneficial effects
Source: J Intensive Care. 2015 Jul 3;3(1):31. doi: 10.1186/s40560-015-0097-0 (PMC4490727; doi:10.1186/s40560-015-0097-0)
Supplement: Additional file 8: — Pulmonary microbiological findings and therapy in patients with isolated pulmonary Candida spp. colonization without pre-existing pneumonia during observation period (cohort 2). Different antifungal drugs and antibiotics that were given and Candida spp., Aspergillus and any bacterial findings during observation period are shown. [file 40560_2015_97_MOESM8_ESM.pdf]

Additional file 8. Pulmonary microbiological findings and therapy in patients with isolated pulmonary *Candida spp.* colonization without pre-existing pneumonia during observation period (cohort 2).

	Antifungal therapy (n=44)	No antifungal therapy (n=131)	p-value
Candida spp., n (%)			
albicans	34 (77.3%)	114 (87%)	0.148
glabrata	16 (26.4%)	24 (18.3%)	**0.021**
tropicalis	6 (13.6%)	7 (5.3%)	0.094
krusei	3 (6.8%)	1 (0.8%)	**0.05**
others[1]	4 (9.1%)	3 (2.3%)	0.068
**Co-infection with** *Aspergillus*, n (%)	1 (2.3%)	1 (0.8%)	0.441
**Any new bacterial finding**, n (%)	23 (52.3%)	68 (51.9%)	1
Gram positive	8 (18.2%)	17 (13%)	0.456
Gram negative	21 (47.7%)	58 (44.3%)	0.728
E. Coli	5 (11.4%)	11 (8.4%)	0.553
Pseudomonas aeruginosa	5 (11.4%)	7 (5.3%)	0.18
Klebsiella pneumonia	1 (2.3%)	8 (6.1%)	0.453
Enterobacter species	4 (9.1%)	5 (3.8%)	0.232
Other gram negative bacteria[2]	6 (13.6%)	27 (20.6%)	0.377
multi drug resistant pathogens	3 (6.8%)	3 (2.3%)	0.168
**Antibiotic treatment**, n (%)	44 (100%)	99 (75.6%)	**<0.001**

[1] *Candida famata, lusitaniae* and *parapsilosis.*
[2] *Stenotrophomonas, Proteus mirabilis, Serratia marcescens, Citrobacter koseri, Actinetobacter baumanii, Proteus vulgaris, Citrobacter freundii, Morganella morgannii,*
