# Supplementary material for: Antifungal therapy in patients with pulmonary Candida spp. colonization may have no beneficial effects
Source: J Intensive Care. 2015 Jul 3;3(1):31. doi: 10.1186/s40560-015-0097-0 (PMC4490727; doi:10.1186/s40560-015-0097-0)
Supplement: Additional file 9: — Patients with isolated pulmonary Candida spp. colonization without pre-existing pneumonia—stepwise backwards elimination for survival time (cohort 2). Cox regression analysis for independent impact on survival was performed for potential co-variable (Therapy, SAPS II, SOFA score, Age and Cancer). [file 40560_2015_97_MOESM9_ESM.pdf]

Additional file 9. Patients with isolated pulmonary *Candida spp.* colonization without pre-existing pneumonia - stepwise backwards elimination for survival time (cohort 2).

		Exp(B)	95% CI for Exp(B)		B	Wald	p-value
			Lower bound	Upper bound			
**Step 1**	Therapy	0.854	0.467	1.561	-0.158	0.262	0.609
	SAPS II	1.022	0.993	1.051	0.021	2.243	0.134
	SOFA score	1.108	1.011	1.214	0.102	4.766	0.029
	Age	1.014	0.990	1.039	0.014	1.301	0.254
	Cancer	0.995	0.506	1.956	0.005	0.000	0.988
**Step 2**	Therapy	0.855	0.469	1.558	-0.157	0.263	0.608
	SAPS II	1.022	0.993	1.051	0.021	2.254	0.133
	SOFA score	1.108	1.011	1.214	0.102	4.804	0.028
	Age	1.014	0.990	1.039	0.014	1.304	0.253
**Step 3**	SAPS II	1.020	0.992	1.047	0.019	1.977	0.160
	SOFA score	1.106	1.010	1.212	0.101	4.680	0.031
	Age	1.015	0.992	1.040	0.015	1.574	0.210
**Step 4**	SAPS II	1.027	1.003	1.052	0.027	5.007	**0.025**
	SOFA score	1.096	1.004	1.198	0.092	4.175	**0.041**

Exp(B) – odds ratio, CI – confidence interval, B – Not standardized regression coefficient, Wald – Wald-statistics. SAPS II - Simplified Acute Physiology Score II and SOFA - Sequential Organ Failure Assessment ($p<0.05$) are independent variables that significantly influence the dependent variable (survival time).
