# Supplementary material for: Antifungal therapy in patients with pulmonary Candida spp. colonization may have no beneficial effects
Source: J Intensive Care. 2015 Jul 3;3(1):31. doi: 10.1186/s40560-015-0097-0 (PMC4490727; doi:10.1186/s40560-015-0097-0)
Supplement: Additional file 10: — Patients with isolated pulmonary Candida spp. colonization without pre-existing pneumonia—stepwise backwards elimination for pneumonia-free time (cohort 2). Cox regression analysis for independent impact on newly aquired pneumonia was performed for potential co-variable (Therapy, SAPS II, SOFA score, Age and Cancer). [file 40560_2015_97_MOESM10_ESM.pdf]

Additional file 10. Patients with isolated pulmonary *Candida spp.* colonization without pre-existing pneumonia - stepwise backwards elimination for pneumonia-free time (cohort 2).

		Exp(B)	95% CI for Exp(B)		B	Wald	p-value
			Lower bound	Upper bound			
**Step 1**	Therapy	1.048	0.536	2.046	0.046	0.018	0.892
	SAPS II	0.998	0.968	1.029	-0.002	0.017	0.897
	SOFA score	1.029	0.923	1.146	0.028	0.265	0.606
	Age	1.009	0.983	1.036	0.009	0.454	0.500
	Cancer	0.714	0.263	1.938	-0.337	0.438	0.508
**Step 2**	Therapy	1.034	0.546	1.957	0.033	0.010	0.919
	SOFA score	1.026	0.930	1.131	0.025	0.258	0.611
	Age	1.009	0.984	1.034	0.009	0.444	0.505
	Cancer	0.719	0.267	1.939	-0.330	0.424	0.515
**Step 3**	SOFA score	1.027	0.933	1.130	0.026	0.289	0.591
	Age	1.008	0.984	1.034	0.008	0.437	0.509
	Cancer	0.716	0.266	1.922	-0.335	0.441	0.507
**Step 4**	Age	1.009	0.984	1.035	0.009	0.524	0.469
	Cancer	0.740	0.278	1.968	-0.301	0.363	0.547
**Step 5**	Age	1.009	0.984	1.035	0.009	0.508	0.476

Exp(B) – odds ratio, CI – confidence interval, B – Not standardized regression coefficient, Wald – Wald-statistics. No variable independently influences the dependent variable (pneumonia-free time) ($p>0.05$). SAPS II - Simplified Acute Physiology Score II and SOFA - Sequential Organ Failure Assessment.
